# The Characteristics of Peripapillary Retinal Perfusion by Optical Coherence Tomography Angiography in Tessellated Fundus Eyes

**DOI:** 10.1371/journal.pone.0159911

**Published:** 2016-07-27

**Authors:** Xiaolei Wang, Yingying Zheng, Xiangmei Kong, Li Zhu, Xinghuai Sun

**Affiliations:** 1 Department of Ophthalmology and Visual Science, Eye, Ear, Nose and Throat Hospital, Shanghai Medical College of Fudan University, Shanghai, 200031, China; 2 Key Laboratory of Myopia, Ministry of Health (Fudan University), and Shanghai Key Laboratory of Visual Impairment and Restoration (Fudan University), Shanghai, 200031, China; 3 State Key Laboratory of Medical Neurobiology, Institutes of Brain Science and Collaborative Innovation Center for Brain Science, Fudan University, Shanghai, 200032, China; University of Utah (Salt Lake City), UNITED STATES

## Abstract

**Purpose:**

To evaluate the peripapillary and perifoveal retinal perfusions of young healthy eyes with a tessellated fundus using optical coherence tomography (OCT) angiography.

**Methods:**

Thirty-five Chinese subjects with a tessellated fundus and 35 subjects without a tessellated fundus from a population-based cross-sectional study in Shanghai were included. All participants underwent OCT angiography. The flow index and vessel density were examined in the peripapillary and perifoveal retinal areas, and their relationships with other ocular parameters were analyzed.

**Results:**

In the peripapillary area, the eyes with a tessellated fundus had a lower retinal nerve fiber layer (RNFL) flow index (0.055 ± 0.009 vs. 0.061 ± 0.007, P = 0.006), RNFL vessel density (61.8 ± 7.3 vs. 65.9 ± 5.2, P = 0.010), retinal flow index (0.086 ± 0.010 vs. 0.092 ± 0.008, P = 0.012), and retinal vessel density (83.7 ± 5.0 vs. 86.4 ± 3.7, P = 0.018) than the control eyes, and the difference remained significant even after adjustments were made for gender and RNFL thickness. No difference was found in the perifoveal area. Multivariable linear regression analysis showed that the retinal flow index and vessel density in the peripapillary area were significantly correlated with the tessellated fundus diagnosis (flow index: β = -0.006, P = 0.005; vessel density: β = -2.597, P = 0.006), gender (flow index: β = 0.005, P = 0.019; vessel density: β = 3.129, P = 0.002) and RNFL thickness (flow index: β = 0.000, P = 0.002; vessel density: β = 0.190, P = 0.002). The RNFL flow index and vessel density were significantly associated with the tessellated fundus diagnosis (flow index: β = -0.005, P = 0.005; vessel density: β = -3.572, P = 0.008) and the thickness of RNFL (flow index: β = 0.001, P < 0.001; vessel density: β = 0.421, P < 0.001).

**Conclusions:**

Eyes with tessellated fundus with a relative decreased peripapillary retinal perfusion compared with eyes without a tessellated fundus were observed. The findings whether indicate causality that the reduction in the peripapillary perfusion and the peripapillary atrophy in myopia, need further study.

## Introduction

Worldwide, being one of the leading causes of low vision [1, 2], high myopia is more prevalent in the Asian population, especially in China [3, 4]. Retinal detachment, macular atrophy, macular hole, posterior staphyloma, choroidal neovascularization, and lacquer cracks are commonly found in high myopia. Myopia is commonly characterized by a tessellated fundus, which is an important indicator of retinochorodial changes. Hayashi et al showed that of 276 eyes with a tessellated fundus, one eye (0.4%) to progressed choroidal neovascularization, eight eyes (2.9%) to lacquer cracks, and 28 eyes (10.1%) to diffuse chorioretinal atrophy after long follow-up periods [2]. However, the potential mechanism of the tessellated fundus has not been clarified.

A tessellated fundus is the presence of polygonal dark areas of choroid between choroidal vessels attributed to atrophy of the choroid pigmentation and the retinal pigment epithelium layer [5]. A tessellated fundus is generally accompanied by atrophy of choroidal capillaries, but it is not known whether the retinal capillaries are affected. Many studies using different techniques have shown that a decreased retinal and choroidal perfusion occurs in high myopia [6–10]. Since the tessellated fundi characterize in some high myopia, it is needed to investigate retinal perfusion in eyes with a tessellated fundus, because not only that can provide baseline information on physiological variations, but also that can monitor the chorioretinal perfusion in those eyes.

The novel technology of optical coherence tomography (OCT) angiography makes it possible to visualize ocular circulation even to the capillary level [11]. Previous studies found that OCT angiography offers great repeatability both intra-visit and inter-visit during the detect of the blood flow around the optic disc [12, 13] and good reliability for the observation of retinal perfusion in the region of the macula [14, 15]. This study aimed to explore the relationship between the peripapillary retinal perfusion and the tessellated fundus by OCT angiography.

## Materials and Methods

### Participants

This was a prospective population-based comparative study of healthy volunteer recruited study from May to June 2015. The study was approved by the Ethics Committee of Shanghai Eye, Ear, Nose and Throat Hospital, China, and it was performed in accordance with the tenets of the Declaration of Helsinki. Written informed consent was obtained from all participants and their guardians. The consent procedure was also approved by the Ethics Committee.

Volunteers underwent a complete ophthalmic check to determine with no known eye diseases. Randomly, only one eye of every participant was chosen for further examination. Inclusion criteria were normal eyes, as determined by slit-lamp biomicroscopy, ophthalmoscopy, and OCT; and intraocular pressure (IOP) <21mmHg. Exclusion criteria included: 1) history of intraocular surgery or ocular injury; 2) having other ocular or systemic diseases such as glaucoma or diabetes mellitus which might affect the ocular circulation; 3) medication usage within two weeks of the measurements; and 4) exhibiting any sign of pathological changes in fundoscopy examination (chorioretinal atrophy, lacquer cracks, lattice degeneration, staphylomas, or pavingstone degeneration).

### Examination

All participants were interviewed regarding their medical histories and had a complete ocular examination, including refractive status, measurement of best-corrected visual acuity (BCVA), axial length (AL) using IOLMaster (Carl Ziess Inc., Jena, Germany), IOP using noncontact tonometer measurements (Full Auto Tonometer TX-F; Topcon, Tokyo, Japan), slip-lamp biomicroscopy examination, and fundus examination (TRC-NW200, Topcon), together with measurements of retinal nerve fiber layer (RNFL) and ganglion cell complex (GCC) thickness (RTvue OCT; Optovue Inc., Fremont, CA). The mean spherical equivalence (MSE) was calculated as the sum of the spherical diopter (D) and one -half of the cylindrical dioptric power. IOP, pulse rate (PR) and blood pressure (BP) were measured within 5 minutes after the examination of OCT. BP amplitude was calculated as the systolic blood pressure (SBP) minus the diastolic blood pressure (DBP). The mean arterial pressure (MAP) was calculated with the following formula: MAP = DBP + 0.42 (SBP–DBP) [16, 17]. The ocular perfusion pressure (OPP) was calculated by subtracting the IOP from two-thirds of the MAP [18]. The participants were asked to refrain from consuming alcohol and caffeine and from smoking for 12 hours prior to the study, as alcohol, caffeine, and nicotine have been reported to affect ocular circulation [19, 20].

Using a digital fundus camera (TRC-NW200, Topcon, Tokyo, Japan), the fundi of all participants were taken by the same technician. Then they were classified into non-tessellated or tessellated fundi, by three experienced ophthalmologists (XL, XM, and XH) based on the images presented on the computer screen. The classifications were made independently. At least two out of the three ophthalmologists agreed, the final classification could be confirmed.

### Peripapillary and Perifoveal Retinal Perfusion Measurements Using OCT Angiography

OCT angiography was examined with the spectral domain OCT (RTVue-XR Avanti; Optovue, Fremont, CA, USA). Using a light source (centered on 840 nm, bandwidth of 45 nm), this system gets an A-scan with a rate of 70 kHz scans per second. A B-scan consists of 304 A-scans. Utilizing two repeated B-scans at 304 different raster positions, three dimensional (3D) OCT angiography scans can be obtained. Based on a B-scan frame with a rate of 210 frames per second, each OCT angiography volume scan can be acquired in approximately 3 seconds. Two volumetric raster scans, including one horizontal priority (x-fast) and one vertical priority (y-fast), were obtained consecutively. The volumetric scans were processed by the split-spectrum amplitude-decorrelation angiography (SSADA) algorithm, and any motion artifacts were removed by 3D orthogonal registration and merging of the two scans. The exclusion criterion for OCT angiography scans was signal strength index (SSI) <45. All processing was achieved using Optovue software (Optovue. Inc., software version 2014.2.0.93). One eye of each participant was examined and scanned during the same visit.

**Fig 1:**
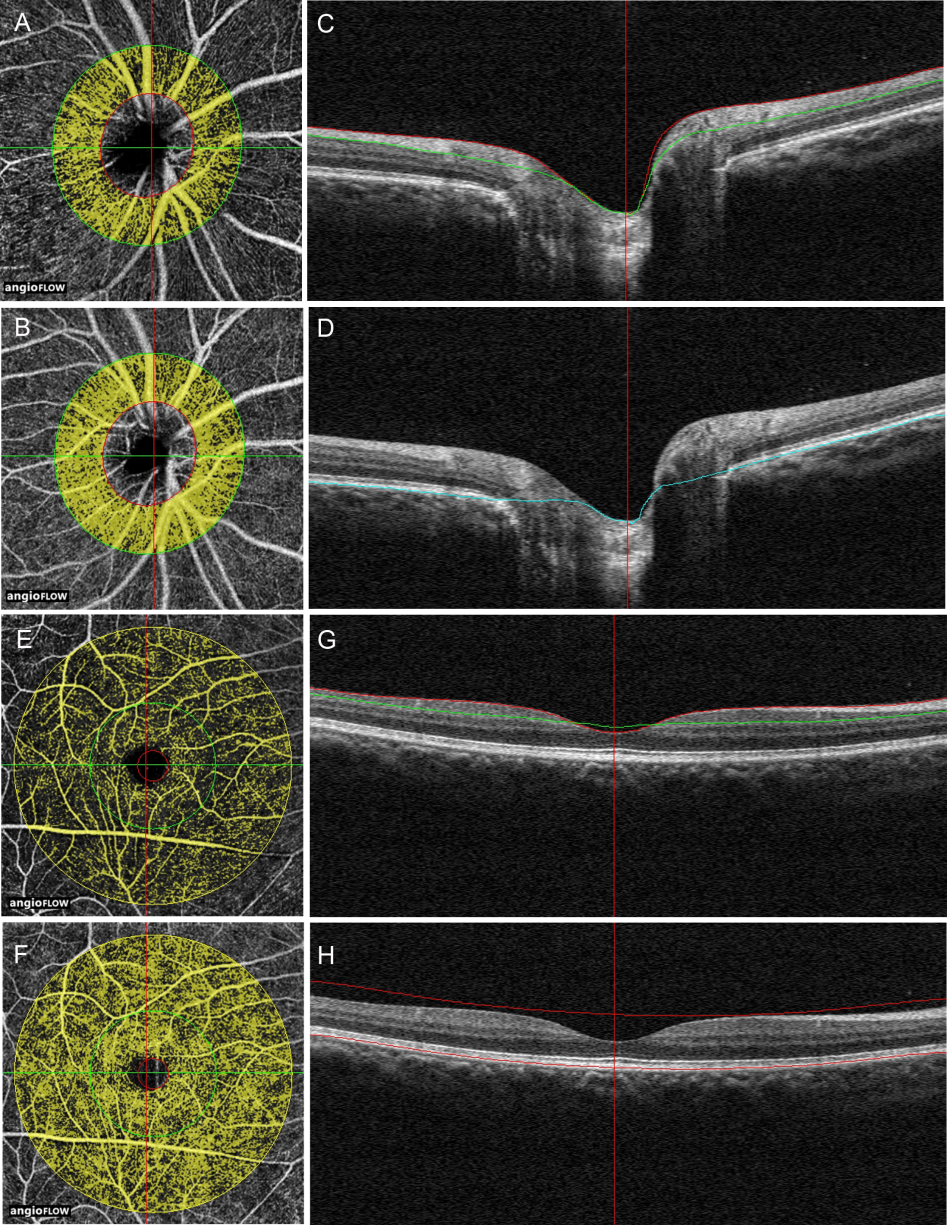
Peripapillary and perifoveal perfusions were measured by optical coherence tomography angiograms of the eye. (A, B) The peripapillary region was defined as a 700 μm wide elliptical annulus extending outward from the optic disc boundary in OCT retinal angiograms. (C) The RNFL was defined from the inner limiting membrane (ILM) to the RNFL on the cross-sectional OCT reflectance. (D) The retinal layer was defined from the ILM layer to the retinal pigment epithelium (RPE) layer on the cross-sectional OCT reflectance. (E, F) A masking procedure of measuring the perifoveal superficial area (E) and the whole retinal (F) perfusion consisted of an annulus defined by an inner diameter of 0.6mm and an outer diameter of 6 mm. (G) The superficial area of the perifoveal was defined as extending from the ILM, with an offset of 3 microns, to the inner plexiform layer, with an offset of 29 microns. (H) The whole retinal area of the perifoveal was defined from the ILM to the RPE layer on the cross-sectional OCT reflectance.

The scan pattern that was used was optimized for SSADA. Each set of scans comprised two 4.5 × 4.5 mm images of the optic disc and 6 × 6 mm images of the perifovea area. The definition of the peripapillary retinal area was described in a previous study [21]. The peripapillary retinal area was defined as a 700 μm wide elliptical annulus extending outward from the optic disc boundary (Fig 1A and 1B). The RNFL perfusion was defined as the RNFL area (Fig 1A and 1C). The retinal pigment epithelium (RPE) was used as the boundary to differentiate the retina from choroid for isolating retinal blood flow (Fig 1D). The perifoveal retinal perfusion was measured using a masking procedure. The masking overlay consisted of an annulus, defined by an inner diameter of 0.6 mm and an outer diameter of 6 mm (Fig 1E–1F). The superficial layer of the perifoveal area was defined as being from the inner limiting membrane, with an offset of 3 microns, to the inner plexiform layer with an offset of 29 microns (Fig 1G). The perifoveal total retinal area was defined as shown in Fig 1H. The perifoveal or peripapillary flow index was defined as the average decorrelation value in the perifoveal or peripapillary region of the en face retinal angiogram. The perifoveal or peripapillary vessel density was defined as the proportion of the total area occupied by vessels. The blood vessels were defined as the pixels with decorrelation values over the threshold in the noise region, which were two standard deviations higher than the mean decorrelation value [22].

### Statistical Analysis

Data analyses were performed using SPSS software (SPSS for Windows, v. 20.0; SPSS, Inc., Chicago, IL). Data were shown as mean ± standard deviation). The independent samples test and the Kruskal-Wallis test were performed to compare the differences between the two groups. A chi-square test was used to analyze the frequency data on gender. Spearman correlation analysis was performed to determine the relationships between the peripapillary perfusion parameters and related factors. Multivariable linear regression analysis was applied to detect the effect of other independent variables on the peripapillary perfusion parameters. Statistically significant was defined as values of P < 0.05.

## Results

Totally 70 eyes of 70 individuals were included in this study: 35 eyes with tessellated fundus, and 35 eyes without tessellated fundus, which were used as controls (S1 Data). The data from both groups were comparable for gender, age, IOP, MSE, AL, DBP, SBP, BP amplitude, MAP, PR, OPP, RNFL thickness, GCC thickness, cup / disc (C/D) area ratio and rim area. The demographic and clinical information is summarized in Table 1.

**Table 1:** Summary of Demographic and Clinical Data of All Subjects.

Characteristics	Non tessellated group	Tessellated group	P value*
	(N = 35)	(N = 35)	
Gender (male:female)	22:13	21:14	**0.806**
Age(yrs)	16.8±0.6	17.1±0.9	0.088
IOP(mmHg)	16.5±3.0	15.3±2.7	0.088
MSE (dioptres)	-5.19±1.49	-5.34±1.07	0.630
Axial length (mm)	25.57±0.76	25.83±0.85	0.190
DBP(mmHg)	68.5±9.5	67.8±7.0	0.711
SBP(mmHg)	112.1±13.4	113.2±12.6	0.735
BP amplitude (mmHg)	43.6±9.3	45.4±10.2	0.443
MAP (mmHg)	86.8±10.4	86.8±8.3	0.993
OPP (mmHg)	41.4±7.2	42.6±6.1	0.448
Pulse rate(bpm)	86.4±13.0	85.3±12.3	0.700
RNFL thickness (μm)	99.4±8.1	98.3±7.8	0.568
GCC thickness (μm)	96.2±5.3	96.4±4.8	0.861
Rim area (mm^2^)	1.4±0.5	1.5±0.3	0.328
C/D area ratio	0.28±0.18	0.24±0.16	0.236

IOP = intraocular pressure; MSE = mean spherical equivalent; DBP = diastolic blood pressure; SBP = systolic blood pressure; MAP = mean arterial pressure; OPP = mean ocular perfusion pressure; RNFL = retinal nerve fiber layer; GCC = ganglion cell complex; C/D = cup/disc. Numbers displayed are mean ± standard deviation.

* All calculated by the Independent Samples Test, except the values in bold, which was calculated by Chi-square test.

**Fig 2:**
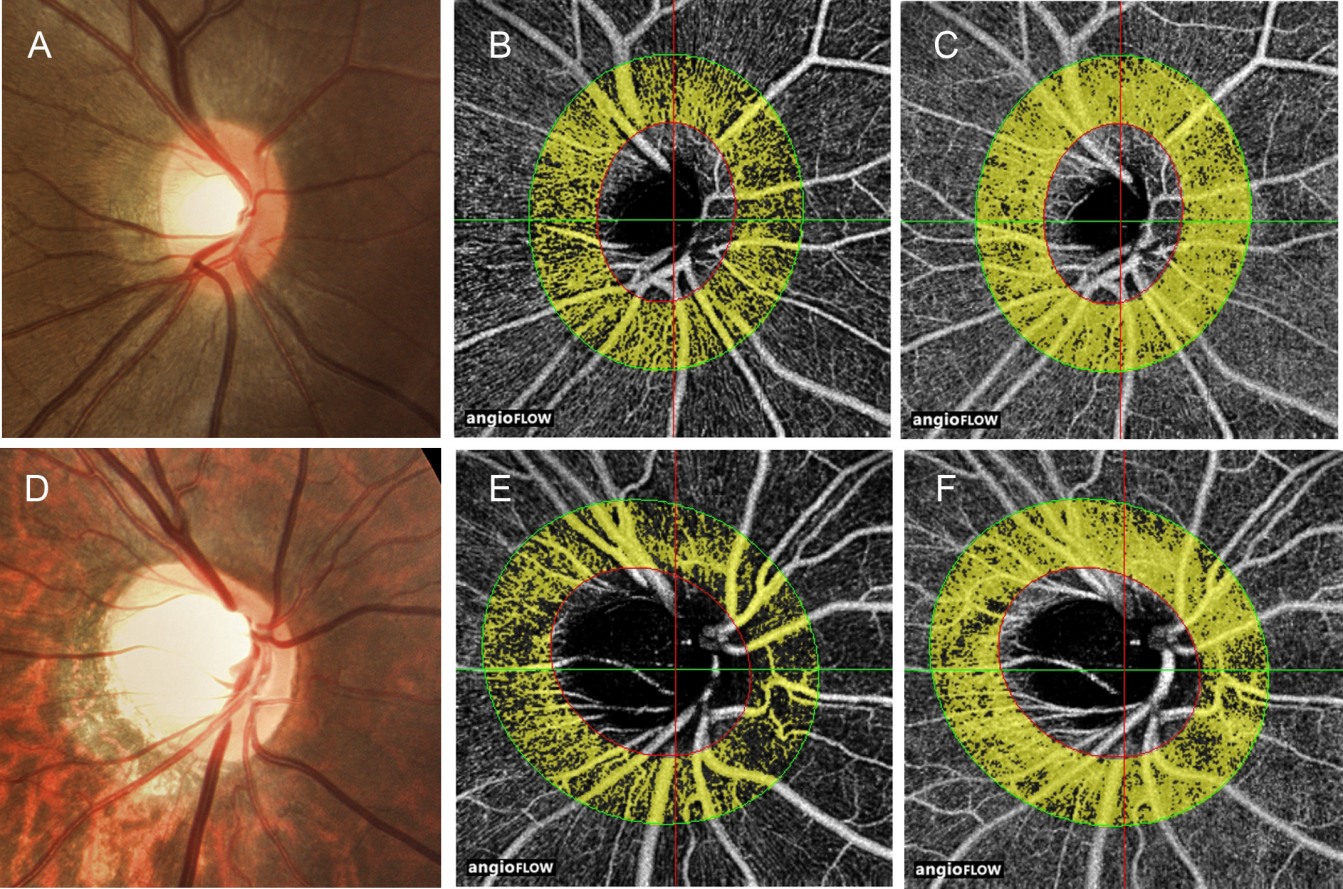
Example of peripapillary perfusion in the non-tessellated and tessellated eyes. Disc photographs (A, D), the RNFL OCT angiograms (B, E) and the whole retinal OCT angiograms (C, F) in the eyes of non-tessellated group (A–C) and tessellated group (D–F). The dense microvascular network of RNFL and whole retina was lower in tessellated group than that of non-tessellated group.

The peripapillary and perifoveal retinal perfusions in the two groups are shown in Table 2. Significant differences were found for the RNFL flow index (0.055 ± 0.009 vs. 0.061 ± 0.007, P = 0.006), RNFL vessel density (61.8 ± 7.3 vs. 65.9 ± 5.2, P = 0.010), retinal flow index (0.086 ± 0.010 vs. 0.092 ± 0.008, P = 0.012), and retinal vessel density (83.7 ± 5.0 vs. 86.4 ± 3.7, P = 0.018) in the peripapillary area (Fig 2). Furthermore, the reduction of retinal perfusion parameters in the RNFL was more predominant than that of the total retinal layer (RNFL flow index: 9.8%; RNFL vessel density: 6.2%; retinal flow index: 6.5%; retinal vessel density: 3.1%). However, no significant differences were found in the perifoveal parameters, including superficial flow index (0.028 ± 0.006 vs. 0.028 ± 0.006, P = 0.711), superficial vessel density (34.0 ± 6.5 vs. 33.1 ± 6.9, P = 0.646), retinal flow index (0.062 ± 0.008 vs. 0.060 ± 0.007, P = 0.256), and retinal vessel density (68.8 ± 6.3 vs. 67.5 ± 5.8, P = 0.234) between the two groups.

**Table 2:** Peripapillary and Perifoveal Retinal Perfusion in two Groups.

Variables	Non tessellated group	Tessellated group	P Value*
	(N = 35)	(N = 35)	
Peripapillary Perfusion			
RNFL flow index	0.061 ± 0.007 (0.045 to 0.077)	0.055 ± 0.009 (0.037 to 0.074)	0.006
RNFL vessel density (%)	65.9 ± 5.2 (51 to 73)	61.8 ± 7.3 (43 to 74)	0.010
Retinal flow index	0.092 ± 0.008 (0.080 to 0.114)	0.086 ± 0.010 (0.067 to 0.108)	0.012
Retinal vessel density (%)	86.4 ± 3.7 (80 to 92)	83.7 ± 5.0 (71 to 93)	0.018
Perifovea Perfusion			
Superficial flow index	0.028 ± 0.006 (0.016 to 0.043)	0.028 ± 0.006 (0.016 to 0.041)	0.711
Superficial vessel density (%)	33.1 ± 6.9 (20 to 48)	34.0 ± 6.5 (20 to 48)	0.646
Retinal flow index	0.060 ± 0.007 (0.046 to 0.077)	0.062 ± 0.008 (0.044 to 0.081)	0.256
Retinal vessel density (%)	67.5 ± 5.8 (56 to 78)	68.8 ± 6.3 (55 to 80)	0.234

RNFL = retinal nerve fiber layer. Numbers displayed are mean ± standard deviation (range).

* Differences between groups were tested with the Kruscal-Wallis test.

The results of the Spearman correlation analysis of the RNFL perfusion and total retinal perfusion parameters are shown in Table 3. The tessellated fundus diagnosis, AL, GCC and RNFL thickness were all significantly related to the RNFL perfusion and retinal perfusion parameters. The gender factor was also significantly related to total retinal flow index and vessel density (flow index: P = 0.034; vessel density: P = 0.002).

**Table 3:** Spearman Correlation Wear Calculated for Variation in Peripapillary Retinal Perfusion.

Parameters	RNFL Flow Index		RNFL Vessel Density		Retinal Flow Index		Retinal Vessel Density	
	r	P Value	r	P Value	r	P Value	r	P Value
Tessellated group vs.Non	-0.334	0.005	-0.311	0.009	-0.302	0.011	-0.284	0.017
Axial length (mm)	-0.245	0.041	-0.277	0.020	-0.350	0.003	-0.387	<0.001
RNFL thickness (mm)	0.524	<0.001	0.508	<0.001	0.446	<0.001	0.380	0.001
GCC thickness (mm)	0.410	<0.001	0.400	0.001	0.247	0.039	0.251	0.036
Gender (male:female)	0.091	0.454	0.167	0.168	0.254	0.034	0.370	0.002
Age (yrs)	-0.028	0.820	-0.046	0.707	0.044	0.718	0.030	0.803
IOP (mmHg)	0.089	0.462	0.080	0.508	-0.050	0.683	-0.004	0.976
OPP(mmHg)	0.017	0.888	0.027	0.822	0.110	0.364	0.023	0.847
Rim area (mm^2^)	0.149	0.218	0.174	0.149	0.022	0.859	0.135	0.266
C/D area ratio	-0.075	0.538	-0.141	0.244	-0.011	0.928	-0.124	0.306

RNFL = retinal nerve fiber layer; GCC = ganglion cell complex; IOP = intraocular pressure; OPP = mean ocular perfusion pressure; C/D = cup/disc.

We performed a stepwise analysis to determine factors mostly associated with the RNFL perfusion and total retinal perfusion parameters in the peripapillary area. In the total retinal perfusion parameters, the model demonstrated the tessellated fundus diagnosis (flow index: β = -0.006, P = 0.005; vessel density: β = -2.597, P = 0.006), gender (flow index: β = 0.005, P = 0.019; vessel density: β = 3.129, P = 0.002), and RNFL thickness (flow index: β = 0.000, P = 0.002; vessel density: β = 0.190, P = 0.002). In the RNFL perfusion parameters, the model demonstrated the tessellated fundus diagnosis (flow index: β = -0.005, P = 0.005; vessel density: β = -3.572, P = 0.008) and RNFL thickness (flow index: β = 0.001, P<0.001; vessel density: β = 0.421, P<0.001) (Table 4).

**Table 4:** Peripapillary Retinal Perfusion and Stepwise Model Multivariable Associations.

Parameters	Retinal Flow Index		Retinal Vessel Density		RNFL Flow Index		RNFL Vessel Density	
	Beta	P Value	Beta	P Value	Beta	P Value	Beta	P Value
Tessellated group, vs.Non	-0.006	0.005	-2.597	0.006	-0.005	0.005	-3.572	0.008
RNFL thickness (mm)	0.000	0.002	0.190	0.002	0.001	<0.001	0.421	<0.001
Gender (male:female)	0.005	0.019	3.129	0.002	/	/	/	/

RNFL = retinal nerve fiber layer.

The stepwise multiple regression only included the tessellated fundus diagnosis, RNFL thickness, and gender for the peripapillary retinal perfusion, and the tessellated fundus diagnosis and RNFL thickness for the peripapillary RNFL perfusion. When multivariate linear regression analysis was used, after controlling for the compounding factors of RNFL thickness and gender, the difference between the tessellated and non-tessellated groups in the peripapillary retinal flow index and vessel density remained statistically significant (retinal flow index: P = 0.005; retinal vessel density: P = 0.006). When controlling for RNFL thickness in the peripapillary RNFL perfusion, the difference between the tessellated and non-tessellated groups in the peripapillary retinal flow index and vessel density also remained significant (RNFL flow index: 0.005; RNFL vessel density: P = 0.008).

## Discussion

It is assumed that a tessellated fundus results from an increase in the visibility of the large choroidal vessel [23]. Recent studies have focused on the choroid, finding a correlation between decreased choroidal thickness and tessellated fundi [24, 25]. To the best of our knowledge, there have been no researches about retinal perfusion of tessellated fundi. OCT angiography is a non-invasive vascular imaging technology that uses intrinsic motion contrast to detect blood flow within the microcirculatory network. The SSADA makes it possible to obtain an OCT angiogram and provide a quantitative flow index and vessel density to evaluate the microcirculation of the retinal and optic nerve [11–13, 15, 22]. OCT angiography has been used to assess retinal perfusion in eye diseases with great intra-visit repeatability and inter-visit reproducibility [11–13]. Our investigation showed the decrease of retinal perfusion in the eyes with tessellated fundus compared those eyes with non-tessellated fundus by OCT angiography. It may be explained that the tessellated fundus is the result of atrophy of the choroid pigmentation and the retinal pigment epithelium layer, and these atrophic changes decrease metabolic need [5].

Additionally, we analyzed the retinal perfusion in different retinal layers and found a trend where the reduction in the RNFL was more predominant than in the total retinal layer. The retinal blood vessels remain in the inner retina including the RNFL and inner plexiform layer. The outer retinal layers are avascular and are supplied by diffusion from the choriocapillaris [26]. Furthermore, the blood supply of the retina consists of both the retinal capillaries and the choroidal capillaries, and since thinning of the choroidal capillaries in the tessellated fundus was observed [25], it could be expected that thinning of the retinal capillaries might also exist.

Although a significant decreased peripapillary perfusion was found between the eyes with a tessellated fundus and without a tessellated fundus, no significant difference was found in the perifoveal area. Yoshihara et al reported that the tessellated changes in the area around the optic nerve were most prominent in young healthy myopia [25]. The reason for the discrepancy may be because we studied younger eyes whose tessellated changes mainly were located in the peripapillary area instead of the perifoveal area. However, further studies for larger samples and a greater age spectrum are needed to examine this issue.

Our results showed that the gender was related significantly to retinal vessel density, not the retinal flow index or RNFL perfusion parameters. This might be related to the lower retinal vessel density found in males. The participants were divided into two groups based on gender (male = 43; female = 27). Significant differences were found only for the retinal vessel density (male: 83.74 ± 4.3; female: 86.44 ± 5.1, P = 0.009). The mechanisms behind these patterns require further study.

The retinal vessel density can be calculated by different methods. Pinhas et al skeletonize the vessels and measure the length of the vessel skeletons over the annulus area to assess the perfused foveal microvascular density. The advantage of this method is that small and large vessels contribute equally to the vessel density metric [27]. In the present study, vessel density was defined as the proportion of the total area occupied by vessels, and the diameters and number of vessels around the disc may affect the vessel density metric. Therefore, the vessel density metric that was used may be biased. However, such a bias was systematic, and comparisons between groups should not be affected because the number of major vessel in the peripapillary area between groups was not significant (non-tessellated group vs. tessellated group: 15.6 ± 1.8 vs16.3 ± 1.7, P = 0.074) in the present study.

The present study has only found a phenomenon of decreased peripapillary retinal perfusion in eyes with tessellated fundus. The sample in this study was not very large and most of the participants were young. We did not assess the peripapillary and perifoveal retinal perfusion parameters in different degrees of tessellation. Further long-term studies with larger samples and a greater age spectrum might tell us more about ocular perfusion in myopic eyes. Furthermore, the entire study population was Chinese, and it is not known whether the results would be the same in other racial groups. However, the observed difference is of interest, and our pilot results emphasize the need for further investigation.

In conclusion, our study showed a reduced peripapillary flow index and vessel density in the retina of eyes with a tessellated fundus by OCT angiography. Whether the reduced peripapillary perfusion in eyes with a tessellated fundus is a cause or result along with its possible role in the pathology of myopia, needs to be clarified in future studies.

## Supporting Information

S1 Data The information of included subjects in this study. (XLSX)

S1 STROBE Checklist STROBE checklist case control. (PDF)
